# Solitary neurofibroma of the floor of the mouth: rare localization at lingual nerve with intraoral excision

**DOI:** 10.1186/s12903-019-0888-0

**Published:** 2019-08-29

**Authors:** Elyette Broly, Benoît Lefevre, Dominique Zachar, Hilal Hafian

**Affiliations:** 1 Faculté d’odontologie, Université Reims Champagne Ardenne, Reims, France; 2 Pôle de Médecine Bucco-Dentaire, Hôpital Maison Blanche, Centre Hospitalier Universitaire, Reims, France; 3 Pôle de Biologie Médicale et Pathologie, Hôpital Robert Debré, Centre Hospitalier Universitaire, Reims, France; 4 Laboratoire de Recherche en Nanoscience (LRN) EA 4682, Université Reims Champagne Ardenne, Reims, France; 5 Department of oral Medicine and Oral Surgery, University of Reims Champagne Ardenne, Maison Blanche Hospital, University Hospital of Reims, 45 rue Cognac Jay, 51100 Reims, France

**Keywords:** Solitary neurofibroma, Lingual nerve, Magnetic resonance imaging, S-100 protein, EMA

## Abstract

**Background:**

Neurofibromas (NF) are benign tumors of the peripheral nerves that are composed of Schwann cells, perineural-like cells and fibroblasts. The differential diagnosis for a solitary intraneural variant of neurofibroma arising in the floor of the mouth is broad and includes a submandibular gland neoplasm and adenopathy, among others. The intraoral approach is the best choice for a medium-sized lesion.

**Case presentation:**

We report a rare case of a solitary neurofibroma of the floor of the mouth in a 31-year-old male. The patient consulted the dental emergency department for acute pain of the left mandible. Systematic clinical examination revealed the presence of a mass in the left mouth floor. The panoramic x-ray was not conclusive and the magnetic resonance imaging (MRI) revealed a well-defined soft tissue lesion with homogenous isosignal intensity on the T1-weighted image, high intensity signal on the T2-weighted image and heterogeneous enhancement following contrast-enhancement on the T1-weighted Fast Sat image. The surgical excision of the soft-tissue neoplasm was accomplished by an intraoral approach. The specimen was sent for histopathologic analysis and Immunohistochemical studies which confirmed the diagnosis of a myxoid predominant intraneural solitary neurofibroma.

**Conclusion:**

The diagnosis of neurofibroma was confirmed by histopathological evaluation and immunohistochemical studies which also excluded other entities in the histopathologic differential diagnosis including schwannoma and a malignant peripheral nerve sheath tumor among other. Localized (solitary) neurofibromas most often occur as sporadic lesions, however; diagnosis of a solitary neurofibroma prompts clinical evaluation to exclude the remote possibility of neurofibromatosis. The purpose of this case report is to raise awareness of the uncommon presentation of neurofibroma and to document the successful management of such a lesion using an intraoral approach.

## Background

Neurofibroma is a benign peripheral nerve tumor composed of a variable mixture of Schwann cells, perineurial-like cells and fibroblasts, as well as cells with intermediate features between these cell types. It is the most common peripherical nerve sheath tumor, and the frequency of solitary sporadic neurofibromas occurring in the oral cavity is reported to the approximatively 6.5% but [1–5]. The usual Clinical presentation of the oral neurofibromas is a discrete, non-tender, submucosal masses. The tongue and buccal mucosa are the more frequent sites and the posterior mandible is the most common intraosseous location [6–8]. Neurofibromas can occur as solitary lesions, as part of a generalized syndrome of neurofibromatosis (von Recklinghausen’s disease) or very rarely as multiple neurofibromas with no association with localized or solitary growths, diffuse discrete multiple nodules and plexiform types [9].

The frequency of isolated neurofibromas unassociated with neurofibromatosis in the oral cavity is uncertain; 4 to 7% of patients affected by neurofibromatosis display oral manifestations [10]. We report a case of an isolated neurofibroma of the floor of the mouth, in the absence of syndromic neurofibromatosis, fortuitously discovered during a systematic clinical examination.

## Case presentation

A 31-year-old man consulted the department of dental emergencies of the hospital for acute pain of the left mandible. Dental examination revealed irreversible pulpal involvement related to a carious lesion of the second premolar. The treatment consisted of opening the pulp chamber and treatment with a sedative pulp medication.

**Fig. 1:**
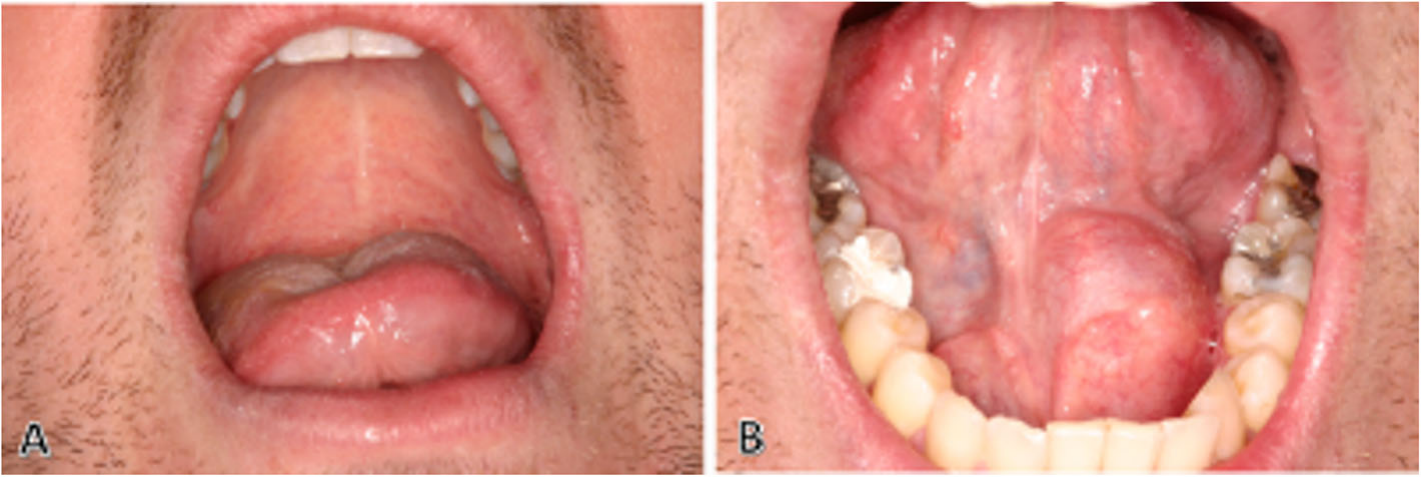
**a**, Intraoral view showing passive rise of the tongue at rest predominantly left side. **b**, Elevation of the homolateral oral floor

Systematic clinical examination of the oral cavity showed tongue elevation (Fig. 1a), predominant on the left side, which revealed the presence of a mass in the homolateral mouth floor (Fig. 1b). The appearance of the mucosa of the floor of the mouth was without abnormality and it was not adherent to the underlying mass. The bi-digital palpation of the left mouth floor revealed a firm, elastic, mobile, oval mass. Its size was estimated at 40 mm × 20 mm and its orientation was anteroposterior with a deep insertion at the rear of the oral floor. The examination of the skin of the head and neck was without abnormality, and the palpation of the lympho-nodes of the cervicofacial areas did not reveal lymphadenopathy. The patient was symptom-free and reported no history of swelling, pain or paresthesia. Considering the clinical characteristics, the differential diagnosis can be made, with a ranula, with a benign lesion of the floor of the mouth such as a dermoid cyst, a fibroma, a lipoma and neurogenic lesion, with an benign tumor of an accessory or submandibular salivary gland such as the pleomorphic adenoma, and with a low-grade malignancy such as mucoepidermoid carcinoma. Finally, the clinical differential diagnosis with lymphadenopathy cannot be excluded.

Previous medical history consisted of a non-displaced traumatic fracture of the 2nd and 3rd left metacarpals and an episode of dry pericarditis. His surgical history was a road accident with head trauma (Glasgow 5), with a bifocal mandibular fracture, a fracture of the left clavicle, a double fracture of the left radius and ulna and a pilonidal cyst. The four third molar teeth were extracted for an orthodontic treatment. No allergies were known to the patient.

**Fig. 2:**
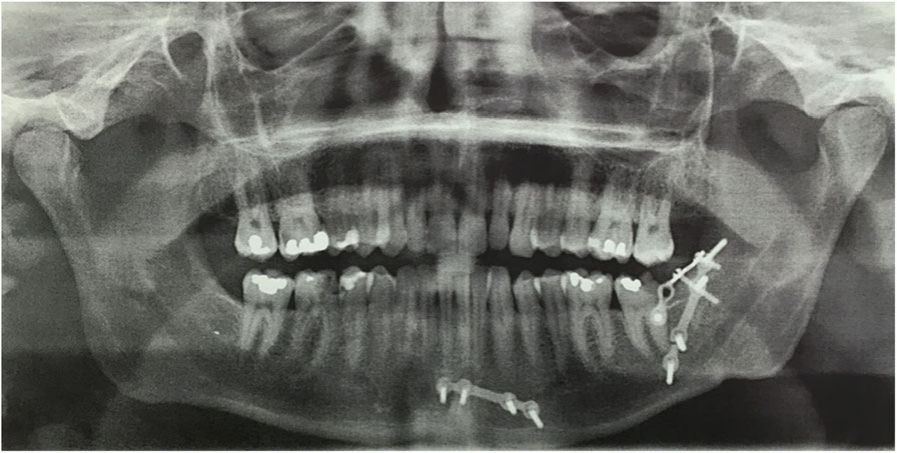
Preoperative panoramic X-ray. The trabecular bone of the region of teeth 33–37 is normal. No alteration of the periodontal ligament space is apparent. The inferior alveolar canal retains normal characteristics. Note the osteosynthesis plates in the paramedian and retromolar position of the left mandible

The panoramic x-ray of the jaws did not show a pathological image, the presence of two plates of osteosynthesis was noted located in the posterior and symphysis of the left mandibular bone. We also noted numerous carious lesions (Fig. 2).

**Fig. 3:**
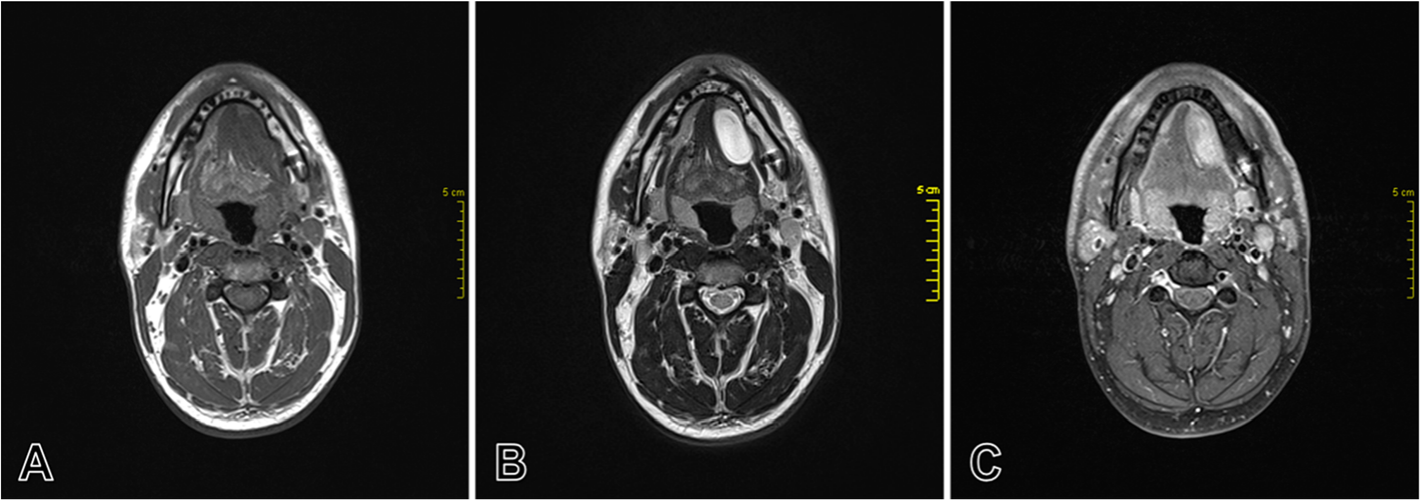
Axial MRI images of the floor of the mouth showing a mass on the left side. **a**, T1-weighted image shows low homogeneous signal of the mass. **b**, T2-weighted image shows high signal intensity of the mass. **c**, Gadolinium-enhanced T1-weighted Fast Sat image shows a heterogeneous enhancement of the signal intensity

Magnetic resonance imaging revealed a well-defined soft tissue lesion of 18 mm × 30 mm × 35 mm in left mouth floor, pushing the submandibular gland forward. The mass showed a homogenous isosignal intensity signal on the T1-weighted image (Fig. 3a), high intensity signal on the T2-weighted image (Fig. 3b) and heterogeneous enhancement following contrast-enhancement on the T1-weighted Fast Sat image (Fig. 3c). The sialograph sequence did not show an increase in the signal in the lesion. The image of Wharton’s canal showed it to be uninterrupted and slightly dilated upstream. The contralateral submaxillary gland and the parotids were normal, incompatible with a salivary cyst.

**Fig. 4:**
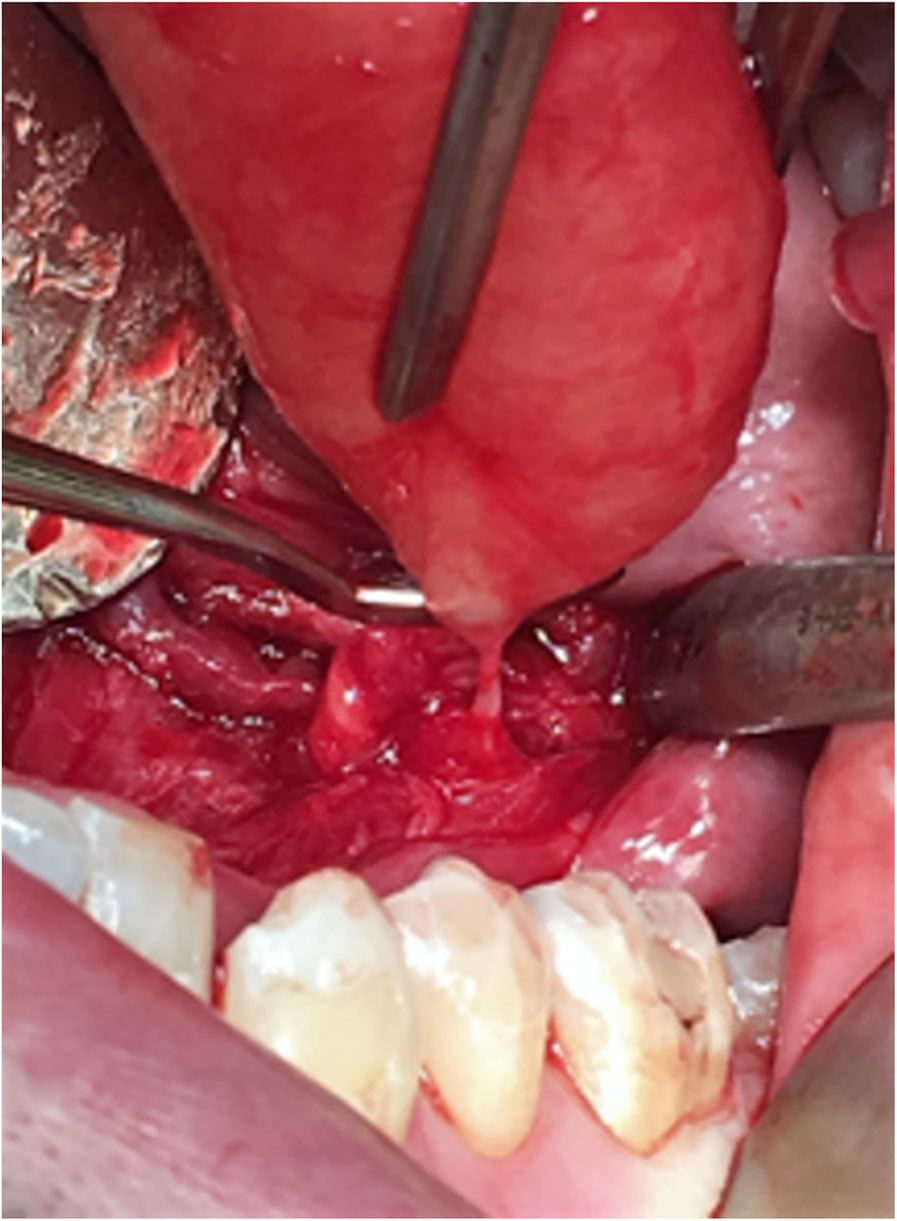
Intraoral view at the end of the dissection. The mass in the posterior region of the floor of the mouth appears to originate from a branch of the lingual nerve

The lesion was completely removed by an intraoral approach under general anesthesia. It was well delimited, cleavable and independent of the submandibular glands. Complete dissection of the lesion showed a millimetric posterior pedicle, evoking a benign nervous tumor having developed on the lingual nerve (Fig. 4). The postoperative sequences were simple, consisting of transient paresthesia of the floor of the mouth and the tip of the left tongue, which resolved in three weeks.

**Fig. 5:**
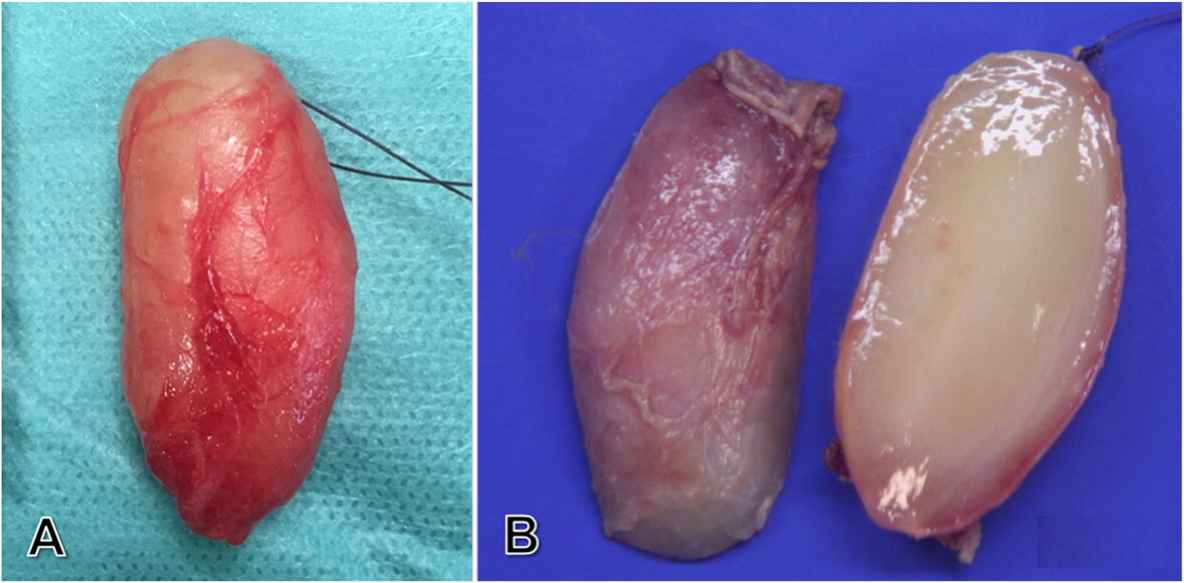
**a**, Gross view of the excised specimen showing a well-circumscribed, oval, yellowish-pink mass measuring 47 mm × 18 mm. **b**, Longitudinal cross-section of formalin fixed excised soft tumor showing homogenous, shiny, myxoid, translucent and light yellow

Gross examination of the excised specimen showed it to be a well-defined, oval, soft tissue mass, weighing 9 g and measuring 47 mm × 18 mm, with a yellowish-pink appearance (Fig. 5a) and a consistency that varied from soft to firm. After fixation, the cut surface of an axial cross-section of the excised mass appeared solid, homogenous, shiny, myxoid, translucent and light yellow (Fig. 5b).

**Fig. 6:**
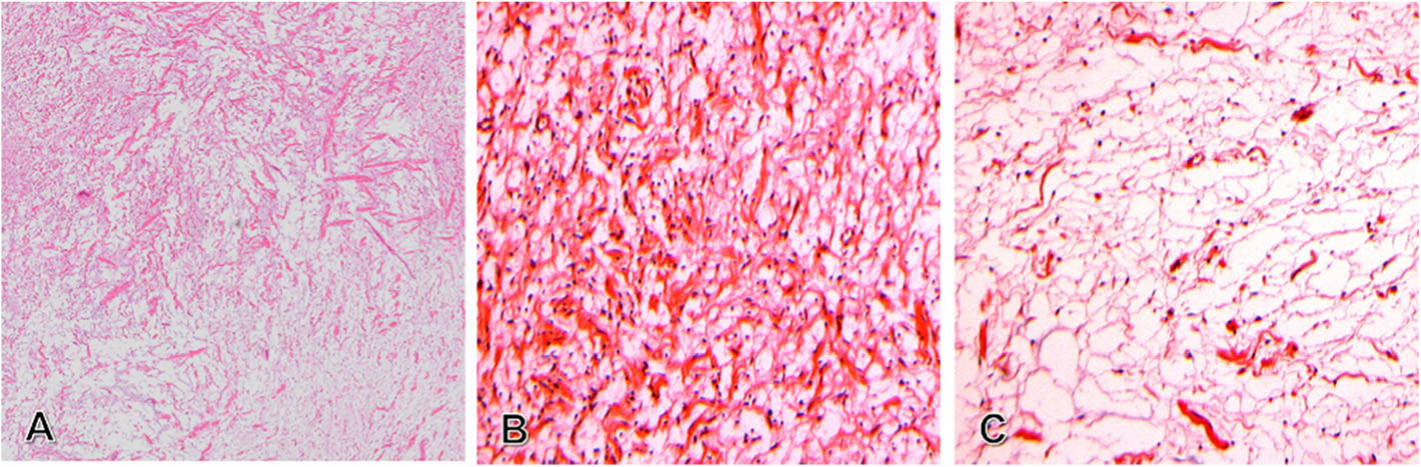
Photomicrograph of the center of the tumor showing a heterogeneous composition (H&E), with more cellular areas containing wavy bundles of fibrous collagen (panel **a**, upper left corner, original magnification 25X, and panel **b**, original magnification 200X) and less fibrous, less cellular areas with a more myxoid background (panel **a**, lower right corner, original magnification 25X, and panel **c**, original magnification 200X)

**Fig. 7:**
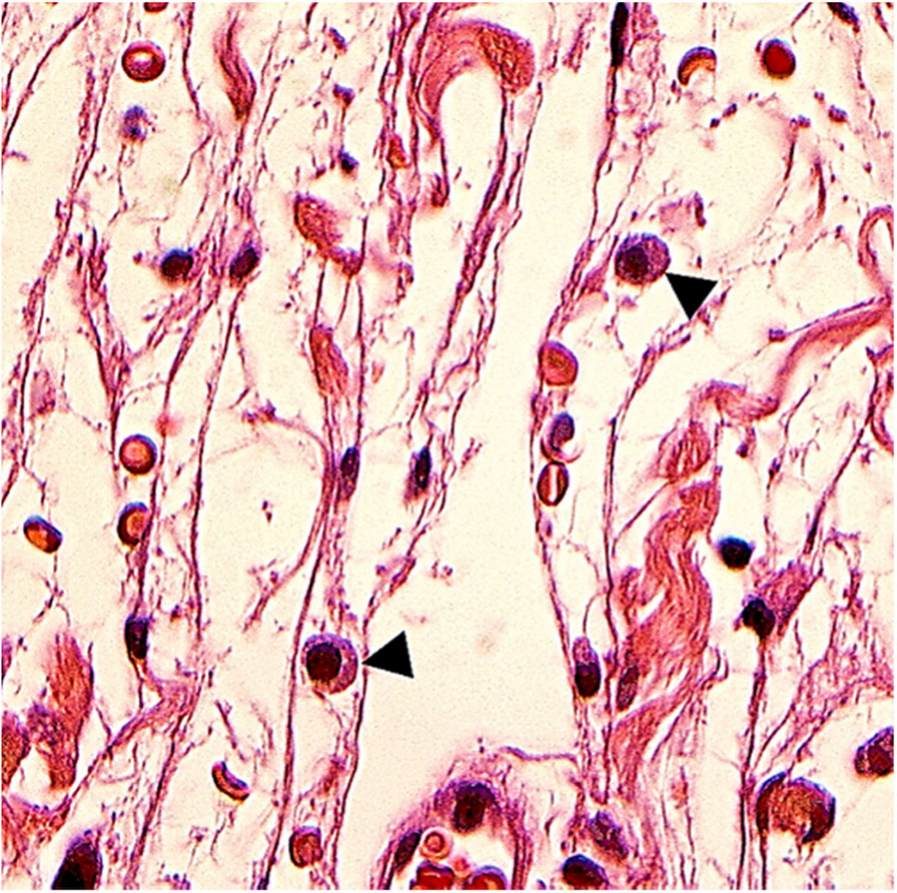
Photomicrograph of the peripheral area of the tumor showing mast cells (black arrow heads) (H&E, original magnification 63X)

Microscopic examination of hematoxylin and eosin (H&E) stained soft tissue sections showed variable staining (Fig. 6a), with a more cellular and fibrous area (Fig. 6b) and a cell-poor area with a myxoid background (Fig. 6c). The lesion consisted of fragmented wavy dense bundles of collagenous fibers spaced on a cell-poor myxoid background, punctuated by scattered small lymphocytes and rare mast cells at the periphery of the lesion (Fig. 7). The wavy neuroid elements were small with low cellularity. There was no nuclear atypia or mitotic activity. Tumor tissue within the thin, laminated perineural sheath was evident within the fibrous adipose connective tissue. There was no salivary gland or salivary canal. The excision was complete.

**Fig. 8:**
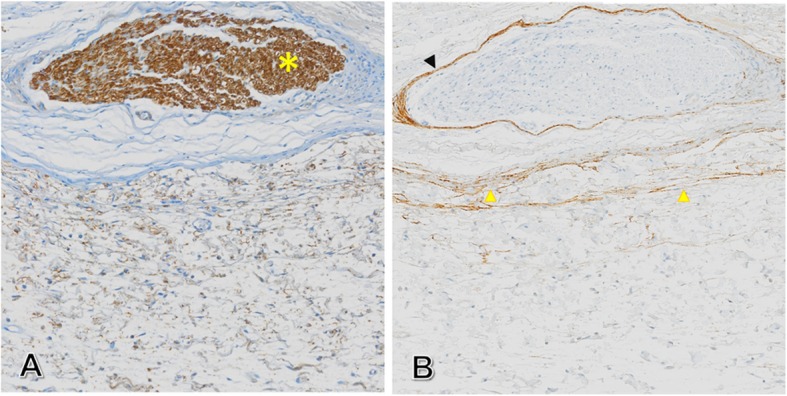
Immunohistochemical studies. **a**, Immunolabeling for S-100 protein: yellow Asterix (*) denotes strong stained nerve at the periphery of the lesion while, neurofibroma tissue shows very weak immunoreactivity. **b**, Epithelial membrane antigen (EMA) immunostaining of laminated external sheath of the tumor, consisting, in part, of the weakly (EMA)-immunoreactive and residual perineurial sheath at the periphery of the neurofibroma (yellow arrow heads). The perineurium of a normal peripheral nerve is thin and non-stratified (black arrow head)

The tumor section showed low positive reactivity to S-100 relative to the normal nerve (Fig. 8a) localized on the surface of the tumor. We observed low positive reactivity to epithelial membrane antigen (EMA) in normal peripheral nerves and the thin, laminated peritumoral sheath (Fig. 8b). The final diagnosis, based on both histopathological and immunohistochemical analysis, was consistent with the clinical diagnosis of an intraneural neurofibroma located on the floor of the mouth having developed on a small branch of lingual nerve.

## Discussion

Neurofibroma of the floor of the mouth is an extremely rare extra-osseous localization of this tumor in the oral cavity. To date, 26 reported cases of neurofibroma of the oral cavity have been reported in the English literature with various localizations (Table 1). Five were localized in the gingiva [6, 11–14], five in the palate [15–19], four on the lip [20–23], seven on the tongue [5, 24–29], one on the cheek [30], four on the mouth floor and in the submandibular region [2, 3, 31, 32].

**Table 1:** Solitary neurofibromas of the oral cavity identified in the literature (26 cases) including year country, lesion site and management

First author, (year) [ref]	Country	Lesion site	Management
Wang, (2006) [2]	Taiwan	Floor of the mouth	Surgical excision
Maruyama, (2011) [3]	Japan	Floor of the mouth	Surgical excision
Mahmud, (2016) [5]	India	Tongue	Surgical excision
Depprich, (2009) [6]	Germany	Gingiva	Surgical excision
Dayal, (2014) [11]	India	Gingiva	Surgical excision
Ohno, (2010) [12]	Japan	Gingiva	Surgical excision
Alatli, (1996) [13]	Turkey	Gingiva	Surgical excision
Richards, (1983) [14]	USA	Gingiva	Surgical excision
Costa, (2012) [15]	Brazil	Hard palate	Surgical excision
Mazzoleni, (2009) [16]	Italy	Hard palate	Surgical excision
Johann, (2006) [17]	Brazil	Hard palate	Surgical excision
Choi, (2011) [18]	Korea	Soft palate	Surgical excision
Pollack, (1990) [19]	USA	Hard palate	Surgical excision
López-Jornet, (2010) [20]	Spain	Upper lip	Surgical excision
Zwane, (2011) [21]	South Africa	Lip	Surgical excision
Traiger, (1966) [22]	USA	Upper lip	Surgical excision
Cuevas-Mons, (1993) [23]	Spain	Lower lip	Surgical excision
Madhumita, (2007) [24]	India	Tongue	Surgical excision
Surwald, (2002) [25]	United Kingdom	Tongue	Surgical excision
Acampa, (1990) [26]	Italy	Tongue	–
Roy, (1965) [27]	India	Tongue	–
Lahoz Zamarro, (1990) [28]	Spain	Tongue	Surgical excision
Sakata, (2012) [29]	Japan	Tongue	Surgical excision
Kınış, (2013) [30]	Turkey	Cheek	Surgical excision
Al-Omran, (2006) [31]	Kingdom of Bahrain	Floor of the mouth	Surgical excision
Chao, (2015) [32]	Taiwan	Floor of the mouth	Surgical excision

All of these lesions developed on the terminal branch of the fifth cranial pair. Neurofibroma of the lingual nerve is extremely rare. Such a lesion typically originates from one of several terminal branches of the lingual and hypoglossal nerves, but it is often difficult to determine the specific origin of the involved nerves. The most common clinical expression is a hypertrophied tongue and fungiform papillae enlargement, particularly in neurofibromatosis type 1 (NF1). However, syndromic manifestations are often associated with other clinical symptoms, such as a large number of both cutaneous and subcutaneous neurofibromas, including more than 10 flat, brown-pigmented café-au-lait spots on the trunk and upper arms [33, 34].

Solitary oral neurofibromas consist of a slow-growing, sometimes painful, submucosal mass [29]. Neurological disturbance is very rare with only a single case of lingual neurofibroma with dysesthesia having been reported in the literature with a diagnosis of neurofibroma confirmed histologically after surgical biopsy of the tongue. The sensory disorders suggest development of the lesion on the sensory nerve, which innervates the posterior third of the tongue [35].

Our knowledge only four cases of neurofibromas located on the oral floor has been reported in the literature, with a presentation as a submandibular mass associated with an elevation of the mouth floor, for three cases of particularly large neurofibromas [3, 31, 32], and one case with large submandibular swelling with latero-cervical progression [2]. In our case, no clinical features, pain or neurological disturbances were reported by the patient. Only a discrete elevation of the mouth floor was seen at the physical examination and moderate size lesion is fortuitously discovered occurring the bi-digital palpation. Floor of the mouth is a challenging site for the diagnosis of a wide variety of lesions that extend from benign to malign lesions. A careful physical examination is a crucial step in the differential diagnosis. Localization, clinical appearance, consistency and adherence to adjacent structures can guide the diagnosis. In our case, the mass was not depressible, not fluctuating, and the patient never presented an acute episode or a functional sign, which is not in favor of a ranula or a dermoid cyst. The firm consistency, the oval and regular shape suggest a benign lesion such as lipoma, a fibroma or neural lesion. However, isolated lymphadenopathy, benign or low-grade malignancy tumor of an accessory or submandibular salivary gland, cannot be ruled out. Diagnostic imaging is an important step in diagnostic orientation and to guide management. MRI is the best radiological examination for the exploration of neurogenic tumors, especially localized to the floor of the mouth. Neurofibromas usually exhibit low to intermediate signal intensity on T1-weighted images and high signal intensity on T2-weighted images, which can be heterogeneous or homogeneous, depending on the composition. A low intensity signal is observed for more fibro-collagenous components and high intensity signal for more edematous and myxoid components. On contrast-enhanced T2-weighted images, the neurofibromas exhibit a well-circumscribed peripheral limits with high signal intensity [36–38]. In our case, T2-weighted images shown heterogeneous tissue appearance with high-intensity signal, these features are in correlation with the myxoid, edematous and weakly cellular aspects observed in histopathological examination. It is possible to suggest neurogenic tumor diagnosis, particularly the typical neurofibroma, based on MRI findings, but differential diagnosis remains difficult to make with other neurogenic lesions such as schwannomas or non-neurogenic lesions such as hemangiomas, lymphangiomas or fibro-myxoid lesions. Clinical examination is, therefore, an important step in the diagnostic orientation, particularly in the oral cavity, which is easily accessible to physical examination.

Without treatment, the mass will increase slowly and painlessly and may cause obstruction of the upper aerodigestive tract. Sarcomatous malignant transformation is possible, especially in Von Recklinghausen Neurofibromatosis. Surgical excision is the standard treatment for neurofibromas. Complete excision of many neurofibromas of the head and neck requires the sacrifice of cranial nerves, subsequently causing significant functional deficits of the upper aerodigestive tract or substantial cosmetic deformity. The intraoral approach through the floor of the mouth is the best treatment approach for lesions evolving above the mylohyoid muscle, as already proposed by Maruyama et al. [3]. This approach is the best option when possible, such as the treatment of dermoid cysts, which frequently occur in the floor of the mouth [39], as it does not leave sequelae, especially scarring of the skin.

Most often, the nerve on the which the tumor is developed is difficult to identify, even if though the intraoral neurofibromas develop most frequently from the branches of the fifth cranial nerves, or more rarely from seventh cranial nerves. After dissection of the mass from connective tissue of the floor of the mouth, we unexpectedly found that this lesion came from the principal trunk of the lingual nerve without any apparent supply vessels (Fig. 4). On this point, our observation was very similar to that reported by Wang et al. [2], who correctly identified the origin of a more posterior and less-evolved neurofibroma in the submandibular space, the mass dissection had been performed by an external cervical approach. In both cases, complete surgical intervention allowed correct identification of the trunk or collateral branch of the lingual nerve as the origin of the tumor and constitutes the best method to provide the most accurate diagnosis.

Histologically, most neurofibromas are circumscribed and non-encapsulated tumors, composed of well-spaced, spindle-shaped cells with elongated, thin nuclei and scant cytoplasm, surrounded by a collagenous matrix situated in the myxoid stroma. In our case, the background was mostly myxoid, with poor cellularity, confirmed by weak histochemical immunoreactivity of the Schwann cells for S-100 protein. Endoneurial and perineurial cells and nerve sheath fibroblasts were S-100 negative [1, 9], whereas there was EMA-antigen immunoreactivity of the perineurial sheath, in particular the residual perineurial sheath, underscoring the localized intraneural form of neurofibroma [8]. The localized intraneural neurofibroma is the most common anatomical variant, it is assumed be neurofibroma confined to a single nerve. Its progressive intraneural growth results in segmental, fusiform nerve enlargement. it may affect any nerve, spinal or cranial, from level of the root to the smallest branches. Localized intraneural form is second in frequency to cutaneous lesions but far less common that the localized cutaneous neurofibroma, this anatomical variant does in general no underlying nerve in appearance, since la proliferation is completely extra neural without perineurium which surrounding the lesion.

The World Health Organization [40] classifies neurofibromas together with schwannomas among peripheral nerve sheath tumors. The exact etiology of solitary neurofibromas remains unknown. Solitary neurofibromas have been postulated to be hyperplasic hamartomatous malformations, rather than a neoplastic disease. A solitary giant neurofibroma of the mental nerve occurring three months after local trauma was reported in a young patient [41]. In our case, it developed on the same side of the mouth as a mandibular fracture with osteosynthesis surgery. The lingual nerve trauma could be suspected as a causal event given the history of ipsilateral mandibular fracture. The surgical treatment with complete excision had been performed successfully and without recurrence. On our case, no recurrence was noted after three years of follow-up, supporting that the lesion was completely removed. In addition, the patient has no neurological sequelae or aesthetic damage, given the intraoral surgical approach.

## Conclusion

Neurofibromas of the floor of the mouth are clinically indistinguishable from other benign soft tissue tumors of the oral cavity. This localization is exceptional and confusing with others neoplasms of the submandibular gland and adenopathy, among others. Knowledge of magnetic resonance imaging helped to guide the diagnosis of this solitary and myxoid variant of solitary sporadic neurofibroma. The histopathological analysis and immunohistochemical studies have led to diagnosis. When possible, the intra-oral approach is the best choice for surgical excision without sequelae and without recurrence.

## Data Availability

The datasets used and/or analyzed during the current study are available from the corresponding author upon request.

## Abbreviations

EMA: Epithelial membrane antigen
H&E: Hematoxylin and eosin
MRI: Magnetic resonance imaging
NF: Neurofibroma
NF1: Neurofibromatosis type1
